# South African midwives’ knowledge of the benefits of delayed umbilical cord clamping

**DOI:** 10.4102/safp.v67i1.6154

**Published:** 2025-08-08

**Authors:** Akhona Shosha, Wanda Jacobs, Zelda Janse van Rensburg

**Affiliations:** 1 Department of Nursing, Faculty of Health Sciences, University of Johannesburg, Johannesburg, South Africa

**Keywords:** midwives, knowledge, delayed cord clamping, benefits, midwifery obstetric units

## Abstract

**Background:**

Immediate umbilical cord clamping has been part of the active management of the third stage of labour for centuries. Despite the benefits of delayed cord clamping, immediate cord clamping remains the routine practice in many countries, including South Africa. The aim of this study was to determine South African midwives’ knowledge of the benefits of delayed umbilical cord clamping.

**Methods:**

Employing a quantitative, descriptive, cross-sectional design, 150 midwives from three Midwifery Obstetric Units (MOUs) in a district in one South African province were sampled through a stratified sampling method to complete the survey with an adapted self-administered questionnaire.

**Results:**

The majority of midwives lacked knowledge of the benefits of delayed umbilical cord clamping or were unsure of their knowledge.

**Conclusion:**

There is documented evidence on the benefits of delayed umbilical cord clamping for both mother and newborn. Midwives who participated in the research lacked knowledge of these benefits. Identification of the knowledge deficits of midwives regarding the benefits of delayed umbilical cord clamping may inform future midwifery education pertaining to delayed umbilical cord clamping and its benefits and may improve the practice thereof.

**Contribution:**

This is the first study in South Africa to report on midwives’ knowledge of the benefits of delayed umbilical cord clamping.

## Introduction

‘The ideal time for umbilical cord clamping has been debated among maternal health professionals for decades’.^1^ Immediate or early umbilical cord clamping (ICC) (cutting or clamping the cord in less than 60 s after birth) has been part of the active management of the third stage of labour for centuries.^2^ Despite the evidence that there are no benefits of ICC practice to either the newborn or the mother, it remains the routine practice in many countries.^3^

Immediate or early umbilical cord clamping is included in the active management of labour and involves the administration of an uterotonic drug, immediate clamping and cutting the umbilical cord and delivering the placenta by controlled cord traction.^4,5,6^ The ICC involves clamping and cutting the newborn’s umbilical cord immediately after delivery with no specific timing.^6,7,8^ This newborn is usually immediately managed under a warmer instead of remaining on the mother’s chest, allowing skin-to-skin contact.^5,7^ The ICC may have severe adverse health outcomes for the newborn. This may lead to a loss of cardiac output with a loss of oxygen, loss of stem cells, jaundice, abrupt occlusion of the umbilical arteries and patent ductus arteriosus.^5,9^ As a result of the possible severe adverse health outcomes to the newborn, delayed umbilical cord clamping (DCC) (cutting and clamping of the cord after 60–180 s or more) is currently the practice recommended internationally by the Word Health Organization (WHO) as opposed to the routine practice of ICC.^10,11^

Delayed umbilical cord clamping has been defined as waiting for a minimum of 1 to 3 min after the birth of the newborn or until the umbilical cord has ceased pulsating before clamping and cutting the cord.^10,12^ The WHO guidelines^7^ recommend that DCC should be performed for all newborns, term or preterm, who do not require positive-pressure ventilation. The WHO^7^ further emphasises that the practice of DCC holds the same benefits in both caesarean section and normal vaginal births. A shift from the active management of labour to a physiological third stage of labour, involving DCC should thus rather be considered in all births. The WHO is in support of a physiological third stage of labour as opposed to the active management of labour, and in 2014 the WHO stated that they are no longer in support of ICC.^10^

A physiological third stage of labour ensures a gentle transition from foetus to newborn state, known as the newborn transitional period.^13,14^ A gentle newborn transitional period allows for a stable pulmonary, cardiovascular and cerebral hemodynamic transition.^13^ The WHO further recommends that DCC should be performed for all births and that DCC can be practised while performing the immediate care of the newborn.^7,10^ Uwins and Hutchon^13^ claim that newborns that were afforded a gentle newborn transitional period by performing DCC are often more alert, yet relaxed after birth, and respiratory and cardiovascular stability can be noted.

‘After birth, blood continues to flow in the umbilical arteries from the neonate to the placenta for around 20–25 s’.^1,14,15^ ‘During foetal life, deoxygenated blood is delivered to the placenta via both of the umbilical arteries circulating through the placenta’.^5,13^ ‘Oxygenated blood is then returned to the baby through the umbilical vein’, and newborn transitions occur from the in-utero environment to the first breath of life.^5,13^ If the placental blood flow is abruptly interrupted with ICC at birth, blood is forced back to the placenta instead of to the newborn.^1^ The newborn must then immediately convert from foetal circulation to newborn circulation by closing shunts.^1,15^ However, when DCC is practised, placental transfusion can occur for the newborn. The blood from the placenta fills the ‘newly opened pulmonary circulation, thereby minimising swings in left ventricular output and, consequently, arterial pressure’.^13,15^

Evidence-based practice revealed that there are several benefits in delaying the clamping and cutting of the umbilical cord after birth for newborns.^2,13,16^ The newborn receives an additional 20 mg/kg – 30 mg/kg of iron and an extra blood volume of 100 mL, of which the first 80 mL is delivered within the first minute (40.0% – 60.0% red blood cell volume).^11,17,18^ In addition, the placenta residual volume is reduced to 13.0% – 20.0% when DCC is practised at 1–3 min.^18^ The blood that would have remained in the placenta is therefore given back to the newborn.^18^ The risk of newborn anaemia is thereby reduced, potentially stabilising the newborn’s blood pressure and cardiorespiratory function, and improving the overall health outcomes of the newborn.^2,3,17^ Newborns also ‘gain protected time for the adjustment because circulation from the placenta continues for a few minutes after birth and the newborn continues to obtain oxygen through the umbilical cord’.^1^ Immediate or early umbilical cord clamping deprives the newborn of about ‘30.0% of its circulatory blood volume at the most critical few minutes of his or her entire life leading to potential iatrogenic hypoxia, hypovolaemia, anaemia and low iron-stores’.^3^

The DCC has a major impact on the newborn’s well-being.^18^ Newborns are especially ‘vulnerable to iron-deficiency anaemia because of their increased iron requirements during times of rapid growth, particularly in the first 2 years of life’.^1^ The DCC decreases ‘the risk of anaemia by 47.0% and the risk of iron store deficiencies by 33.0% at ages 2–3 months’.^17^ The DCC also increases the supply of stem cells to the newborn and could be an ‘effective and non-invasive way of transplanting these cells to the infant to prevent and combat neonatal and age-related diseases’.^17^ The DCC has proven to have long-term neurodevelopmental effects on the growing newborn.^3,19^ Newborns ‘with iron deficiency anaemia are more likely to have delayed psychomotor development’.^1^

The DCC decreases the occurrence of red blood cell alloimmunisation for the mother.^1^ There is no ‘risk for the mother with respect to postpartum haemorrhage, increased blood loss at delivery, lower haemoglobin levels, or need for a blood transfusion’.^17^ The WHO also stated that there is no evidence that suggests that ICC is needed for the prevention of postpartum haemorrhage.^7^ The DCC has been proven to reduce morbidity and mortality of both the newborn and mother.^1,3,11^

The practice of DCC is associated with midwives’ knowledge of the risks and benefits of DCC.^3^ Several international studies regarding the knowledge, practices and attitudes of midwives and obstetricians on DCC have been conducted.^3,20,21^ These studies concluded that midwives have varied levels of knowledge regarding DCC,^22,23,24^ which includes knowledge of the benefits of DCC as well as possible related risks, such as the reported susceptibility for jaundice.^9,22,24^ Several studies propose the reason for midwives being more likely to perform ICC instead of DCC, is the need ‘to speed up the third stage of labour and to get the mother and newborn out of the delivery room’.^3,6,21,23^ Other reasons, such as the ‘presence of a neonatologist or paediatrician who is anxious to attend to the neonate, a desire to place the newborn on immediate skin-to-skin contact’ with the mother, neonatal resuscitation and the lack of awareness of the benefits of DCC were cited for midwives not practising DCC.^3,25^

Delayed umbilical cord clamping practice is still not accepted worldwide, as two thirds of birthing units in developing countries still practice ICC.^2^ Even with well documented evidence that DCC has undisputed benefits to the newborn and no added risks to the mother, many midwives still perform ICC.^2^ The first author, a practising midwife with several years of experience in labour and delivery, observed that the practice of cord clamping is not consistently practised, with some midwives practising DCC and others ICC. It was also observed that midwives do not practice DCC consequently and there is also a variation in the timing of the cord clamping among midwives. Some of the midwives vary their own practices of cord clamping. After an informal conversation with several midwives from maternity units throughout the country, the first author concluded anecdotally that it appears as if the practice of umbilical cord clamping is not a standardised practice followed by midwives in South Africa. It also appeared that both ICC and DCC are practised by midwives. It was noticed that neither the ICC nor DCC is practised consistently during the management of the third stage of labour by midwives, and that ICC appeared to be the practice norm. This led the authors to question the inconsistent management of umbilical cord clamping and whether the lack of consistent practice of DCC is related to a possible lack of knowledge of midwives on the benefits of DCC.

A search for studies on the knowledge of midwives on DCC recently conducted in Africa, revealed only one article.^26^ This study was however conducted in Lesotho and not in South Africa. No other articles related to research on the knowledge of South African midwives on DCC could be found.

The aim of the study was to determine midwives’ knowledge of the benefits of DCC in Midwifery Obstetric Units (MOUs) in a district in one of the provinces in South Africa. Understanding the level of knowledge of the midwives on DCC might explain the reluctance to change from ICC practice to DCC or to consistently practice DCC. This may also inform future midwifery education on DCC and assist in developing guidelines and recommendations for implementation of policies and procedures on DCC that may assist in the reduction of iron-deficiency anaemia in newborns and children and the prevention of newborn and maternal mortality.

## Research methods and design

### Study design and setting

A quantitative, descriptive, cross-sectional^27^ design using a survey method by means of a self-administered questionnaire was used in the study. The self-administered questionnaire included questions to determine the knowledge of the midwives on the benefits of DCC. The self-administered questionnaire was adapted from an umbilical cord clamping survey with permission from the authors^6^ of the existing questionnaire to address the purpose of the study. This survey was used to illuminate the specifics relating to the knowledge of midwives on the benefits of DCC.

The study was conducted across three MOUs in a district in one of the provinces in South Africa. Midwifery Obstetric Units are government facilities that are public-serving and situated in each province of South Africa. The survey was conducted in three of the 38 MOUs in the district from February to May 2020. These three selected MOUs service an estimated 231 064 maternity patients per year. All three MOUs render antenatal, intrapartum, labour and postnatal care to women.

### Study population and sampling strategy

The study population consisted of midwives who were involved in antenatal, intrapartum, labour and postnatal care at the time of the research. A stratified sample^27^ was used to recruit midwives working in the three MOUs to participate in the study. The inclusion criteria for the respondents were midwives registered with the South African Nursing Council (SANC), working full-time or part-time in any of the three MOUs, taking part in antenatal care, intrapartum care, labour and postnatal care, and who were willing to participate in the study.

The recruitment of midwives took place at the three MOUs where the researcher held information sessions at the beginning of each shift explaining the research and invited midwives who met the inclusion criteria to respond to the study. Those who were willing to participate in the study gave written consent to complete the self-report questionnaire. A total of 325 midwives were working at the three selected MOUs at the time of the study. The sample of midwives to be included in the study were calculated using an online sampling calculator (https://goodcalculators.com/sample-score calculator) with a 95.0% confidence level, and an error margin of 5.0% from the total population of midwives. A total of 150 midwives (*N* = 150) participated and a 100.0% response rate was received. No identifiable data such as the name of the respondents were collected, in order to maintain anonymity, privacy and confidentiality.

### Data collection and instrument

A paper-based, self-report questionnaire in English was used for data collection. As mentioned, the researcher held information sessions at the beginning of each shift explaining the research and inviting the midwives to participate. Those midwives who consented to participate in the research were given a self-reporting questionnaire, which they could complete at their own pace and which were collected from sealed boxes by the researcher when the researcher met with the midwives of the next shift.

The questionnaire used in this study was adapted from a questionnaire by Stoll and Hutton^6^ with permission from the authors for the purpose of this study. The questionnaire by Stoll and Hutton^6^ consists of 18 questions of which 13 questions placed the emphasis on newborn resuscitation and performing DCC. Five questions on demographic characteristics of the respondents and DCC policy were added to the questionnaire for this study. The 14 questions on resuscitation were adapted to change the focus to the knowledge and practice of DCC instead of newborn resuscitation perse. Six of these 14 questions asked about the benefits of DCC as stipulated in the WHO guidelines, which was the focus of this article.^7^ The adapted self-report questionnaire consisted of 19 close-ended questions and was presented in a multiple-choice question format providing clear answers that were regarded to be mutually exclusive. Respondents were limited to the choice of only one of four options based on the answer they viewed as most relevant for the posed question. It took the respondents approximately 20 min each to complete the questionnaire.

The adapted self-report questionnaire was divided into two sections exploring respondents’ demographic data in one section and knowledge and practices on DCC in the other section. For the purpose of this article, we only focused on the six questions related to the respondent’s knowledge of the benefits of DCC in the second section.

### Data analysis

After capturing the data from the self-report questionnaires on Microsoft Excel, the data were analysed by a statistician from the university using the IBM^®^ SPSS^®^ (Version 25, IBM Corporation, and New York, United States [US]) statistical software program. The analysed data are presented as descriptive statistics. Frequency distribution was used to determine and visually represent the demographic data.

### Validity and reliability

Prior to the data collection, the tool was reviewed by a statistician from the university. The methodology was applied consistently with standardised processes. The self-report questionnaire was pre-tested with 10 midwives for face validity, reliability and consistency of the questionnaire. The statistician verified the pre-testing results and no changes were made to the questionnaire following the pre-test. The results obtained in this study were found to be comparable with previous international studies that were conducted.

### Ethical considerations

The study was approved by Research Ethics Committee of the Faculty of Health Sciences at the respective university (REC-183-2019). Gatekeeper approval including that from the provincial Department of Health, Protocol and Review Committee, (number GP_202003_042) was obtained before commencing with the data collection in the selected MOUs. The participating midwives signed informed consent before participation and completed the questionnaire voluntarily and anonymously. Respondents were informed of their right to withdraw before submitting the completed questionnaire after which it would not be possible because of the anonymity of the questionnaires. To ensure confidentiality, privacy and anonymity, sealed boxes were placed in each MOU for submission of the questionnaires post completion. No identifying information such as a name was required on the questionnaire ensuring privacy, confidentiality and anonymity. The collected data were kept in a password protected folder on an electronic device for 5 years.

## Results

### Demographics

Responses of a total of 150 (*N* = 150) questionnaires from the three MOUs were analysed. As indicated in Table 1, 35 out of the 150 (23.5%) respondents had 3 to 5 years of experience as a practising midwife, 25 (16.8%) had 5 to 10 years of experience, 36 (24.2%) had 10 and more years of experience as while 53 (35.6%) had less than 2 years of experience as a practising midwife.

**TABLE 1:** Years of experience as a practising midwife (*N* = 150).

Years of experience	*n*	%
< 1	25	16.8
1–2	28	18.8
3–5	35	23.5
5–10	25	16.8
> 10	36	24.2

**Total**	**150**	**100.0**

As depicted in Table 2, the highest qualification of majority (*n* = 117, 78.0%) of the respondents was a nursing degree (general, community, psychiatry and midwifery). While 31 (20.7%) of the 150 respondents held a post basic diploma in midwifery (midwife specialist qualification), only two respondents (1.3%) held a master’s degree in midwifery.

**TABLE 2:** Highest midwifery qualification (*N* = 150).

Highest midwifery qualification	*n*	%
Nursing degree (general, community, psychiatry, and midwifery)	117	78.0
Post basic diploma in midwifery (midwife specialist qualification)	31	20.7
Master’s degree in midwifery	2	1.3

**Total**	**150**	**100.0**

Respondents needed to indicate in which units within the obstetric maternity unit they are working (Table 3). Most of the respondents (*n* = 96, 64.0%) worked in a labour ward, while 54 (36.0%) worked in either the antenatal or postnatal wards, respectively. Majority of the respondents (*n* = 144, 96.0%) were permanent employees in the three selected MOUs. Only six (4.0%) of the 150 respondents were not employed full-time and worked through a nursing agency as part-time staff at the time of the study.

**TABLE 3:** The obstetric maternity unit where the midwives currently work (*N* = 150).

Obstetric maternity unit	*n*	%
Antenatal ward	19	12.7
Labour ward	96	64.0
Postnatal ward	35	23.3

**Total**	**150**	**100.0**

In the questionnaire (Table 4), midwives’ knowledge of the benefits of DCC was put to the test. Four of the six questions in the questionnaire were correctly answered by all respondents (Q1; Q3; Q4; Q5). Between 94 (62.7%) to 118 (78.7%) respondents had some knowledge of the benefits of DCC. In the two questions that were not all answered correctly by the respondents (Q2 and Q6), only 44 (29.3%) and 65 (43.3%) respondents correctly answered these two questions, respectively.

**TABLE 4:** Knowledge of the benefits of delayed umbilical cord clamping (*N* = 150).

Question number	Question or statement	True		False		Not sure	
		*n*	%	*n*	%	*n*	%
1	DCC decreased risk of neonatal hospital stay	103	68.7	26	17.3	21	14.0
2	No developmental benefits of delayed umbilical cord clamping	62	41.3	65	43.3	23	15.3
3	DCC can be done while the newborn is placed on skin-to-skin contact with the mother and performing the immediate care of the newborn at the same time	114	76.0	20	13.3	16	10.7
4	There is an increase in iron-stores for the newborn when practising DCC	118	78.7	12	8.0	20	13.3
5	DCC decreases the risk of respiratory distress in the newborn	94	62.7	24	16.0	32	21.3
6	DCC holds no maternal benefits	73	48.7	44	29.3	33	22.0

DCC, delayed umbilical cord clamping.

## Discussion

A quarter (24.0%) of the respondents indicated that they had been practising as midwives for 10 years or longer, meaning these midwives did not receive any formal education on DCC. According to Mwamba,^28^ DCC was only introduced after 2010 in South Africa therefore only newly qualified midwives (those who started their studies after 2010) received some formal training on DCC.^11^ This means that about three quarters (75.3%) of the respondents were supposed to have received some form of formal training on DCC and would be expected to have sufficient knowledge of the benefits of DCC. The responses indicate that 62.7% to 78.7% of the respondents could answer four of the questions on the benefit of delayed cord clamping correctly while between 56.6% and 70.6% of respondents answered two of the six questions incorrectly. This implies that there is a gap in the knowledge of midwives on the benefits of DCC. Mwamba^28^ suggests that developing an educational programme incorporating evidence-based practices about DCC can result in reducing neonatal anaemia and assist in increasing the midwife’s knowledge of the physiology of foetal transition at birth. Several educational programmes and training are however available in South Africa such as the Essential Steps in Managing Obstetric Emergencies (ESMO),^29^ Basic Antenatal Care (BANC)^30^ and the Immediate Care of the Newborn Resuscitation; however, none of these programmes focus specifically only on evidence-based DCC practice for midwives. The increase in knowledge would impact the incidence of maternal and neonatal anaemia.^28^ This could address the gap in the midwife’s knowledge of the benefits of DCC.

Midwives in South Africa receive their training as a midwife as part of a basic qualification such as the nursing degree, a post graduate diploma, a course work master’s degree (phased out) or research master’s degree. Delayed umbilical cord clamping has been part of the training programme of midwives since 2010,^28^ implying that 75.3% of the respondents received some sort of training on DCC. According to the *Nursing Act*,^28^ a midwife is defined as an individual providing professional care that supports and assists the health care user, particularly the mother and the baby, to achieve and maintain optimum health during pregnancy, the stages of labour and the postpartum period. The *Nursing Act*^31^ classifies the above-mentioned qualifications as a midwifery regime, meaning midwives with any of these qualifications have an influence on the course and management of women’s pregnancy and delivery.^31^ The midwife is involved in all stages of maternity care including antenatal, labour and birth, and postnatal care, as well as the provision of care plans, their implementation and evaluation.^31^ Based on their training and practice according to the *Nursing Act*,^31^ they should have knowledge of and practice DCC. Training usually includes the guidelines from WHO, which were also available from 2014 providing guidelines on the practice of DCC.^7,10^ The National Integrated Maternal and Perinatal care guidelines for South Africa also provide guidance on the practice of DCC.^11^ These guidelines clearly state that DCC applies for both term and preterm births as well as for both vaginal deliveries and caesarean sections.^11^ Rana, Brunello and Malqvist^32^ claim one of the reasons for the lack of DCC practice among midwives is routine practice. The authors claim that midwives tend to clamp the cord either because they were clinically trained to do so or based on organisational procedures and protocols.^32^

Most low-risk pregnant women in South Africa give birth in community-based MOUs run by midwives; intermediate or high-risk pregnant women typically deliver in the hospital.^33^ In addition, there are different referral hospital levels based on the woman’s obstetric risk class (primary, secondary and tertiary hospital).^31^ In this study, a total of 64.0% of the respondents were working in the labour ward in one of the three selected MOUs. Women giving birth in an MOU are classified as low risk. As such, normal vaginal births occur, and there should be no clinical reason why DCC should not be practised.^33^ Regardless of the unit in which the midwife is working, midwives must have knowledge of DCC and be able to practice it,^28^ especially the midwives who received training after 2010. Even when delivery occurs via caesarean section, the midwives should advocate for the practice of DCC based on its documented benefits.^7,10^ Of the 150 respondents, 12.7% indicated they were currently working in an antenatal ward, where midwives educate women about DCC and its benefits. They are still midwives and may need to assist with the birth of a baby at any given time in the labour ward or outside the labour ward, therefore they are expected to have sufficient knowledge of DCC.

On the question of whether DCC decreases the risk of the neonate staying in the hospital, 103 (68.7%) respondents answered correctly that it does indeed decrease the stay of the neonate in the hospital.^34^ An increase in the blood supply to the newborn through placental perfusion leads to the newborn’s increased body weight at birth, which helps in the prevention of hypothermia; this is achieved through DCC and leads to a decreased newborn hospital stay.^34^ DCC resulted in fewer infants being admitted to neonatal intensive care unit (NICU).^34^ Delayed umbilical cord clamping further assists the newborn maintain normal glucose levels because it allows early initiation of breastfeeding, which helps in decreasing newborn hospital stay.^34^ Although three quarters of the respondents received training after DCC was reported to be introduced into training of the midwifes, only 103 (68.7%) answered correctly. Considering the numbers, the expectation would have been that more respondents would have answered the question correctly.

Respondents had to indicate true or false to the statement that DCC held no developmental benefits to the neonate. Sixty-five (43.3%) respondents indicated correctly that the statement was false and that DCC does have developmental benefits to the neonate. Iron-deficiency is a major concern and has been associated with poor neurodevelopment in preschool and school children. The benefit of DCC by increasing iron-stores contributes to the decrease in anaemia^13,18,34^ thus contributing to the development of the neonate. Iron-deficiency in infants impairs neurodevelopment and reduces infant anaemia as a result improving infant and children’s neurodevelopment.^8,18^ The extra placental transfusion through DCC is also effective and safe, and prevents the need for blood transfusions,^18,28,29,30,31,32,35^ which prevents any adverse complications for the neonate impacting on their development. The fact that only 43.3% respondents reported the statement correctly is concerning considering most of the respondents reported that they should have received training on DCC and by implication the benefits thereof.

The respondents had to indicate if the risk of respiratory distress would decrease with DCC. Ninety-four (62.7%) of the responses were correct. The practice of DCC also increases newborn blood supply, leading to a higher respiratory rate and a low risk of developing newborn respiratory disease because of increased oxygen supply from placental transfusion.^32^ During an immediate assessment of newborns where DCC was practised, it was reported that the newborns behaved differently from those subjected to ICC in terms of positive Apgar scoring and easy respiratory effort.^13^ According to available literature^13,36^ DCC decreases the newborn’s risk of respiratory distress and for that reason 62.7% respondents were correct in their response.

The respondents had to indicate if it is true or false that there is an increase in iron-stores for the newborn when practising DCC. In all, 118 respondents (78.7%) were correct by stating it is true. With term newborns, the practice of DCC increases the haemoglobin levels at birth, improving the iron-stores for several months of life, which may have favourable effects on developmental outcomes.^8,35^ Delayed umbilical cord clamping is also associated with significant newborn benefits in preterm newborns, including improved transitional circulation, better establishment of red blood cell volume, decreased need for blood transfusion and lower incidence of necrotising enterocolitis and intraventricular haemorrhage.^18,35^ ‘It is well established that delayed cord clamping improves total body iron-stores for up to 6 months of age and reduces the need for blood transfusion for anaemia’.^12,34^ The improved total body iron leads to improved haemoglobin for 24–48 h of life in term newborns.^18,35^ The benefit for DCC in the increase of iron-stores was confirmed in numerous studies for preterm and full-term babies with the benefits of reducing anaemia observed for 2 to 6 months after delivery.^18^ Conversely, 8.0% of the respondents disagreed that DCC increases the newborn’s iron-stores. The conclusion is therefore that the midwives participating in this research had knowledge of the benefits of DCC on the iron-stores of a newborn.

A total of 114 (76.0%) respondents indicated correctly that DCC can be performed while the newborn is placed in skin-to-skin contact with the mother while performing the immediate care of the newborn at the same time. The immediate management of the healthy newborn and the process of DCC goes together.^7,10^ The WHO guidelines stipulate the following steps for immediate management of the newborn while allowing DCC.^7,10^ The delivery midwife must call out the time of birth to the selected assistant, a colleague pre-selected by the midwife to assist with the delivery process. Following the time of birth, the newborn is placed on the mother’s abdomen or chest to allow skin-to-skin bonding. The midwife then dries the newborn thoroughly with a warm, dry and clean towel while simultaneously assessing the newborn’s breathing. The newborn is covered with a clean, dry cloth to prevent hypothermia while still delaying the cord clamping. Routine care is still performed by the midwife, including the identification of the newborn along with the mother through identification bands. The umbilical cord is then clamped only when the cord has stopped pulsating or when 1–3 min has elapsed after birth.^7,10^ Because of DCC, the incidence of intubation in the delivery room on account of respiratory distress was significantly lower.^5^ An infant who needs resuscitation or complex supported interventions with the umbilical cord attached can be performed safely, is effective and feasible, and DCC is recommended for newborns requiring interventions such as resuscitation with several authors^18,28,29,30,31,32,35^ concluding that clinicians should advocate for the implementation of DCC as part of the resuscitative process.

The respondents had to indicate if it was true or not that DCC held no benefits for the mother. A total of 44 respondents indicated correctly that this statement was false, and that there are benefits for the mother. The International Liaison Committee on Resuscitation as well as the European Resuscitation Council recommend the practice of DCC because of the benefit it has for both the mother and the newborn.^7,10,13,23,37^ The WHO guidelines highlight that one of the reasons for DCC is that it improves maternal and newborn health.^7,10,13,23,36^ Qian et al.^18^ concluded in their synthesis of literature findings on the benefits of DCC that DCC does not appear to increase risk in maternal postpartum haemorrhage and the DCC was feasible from an obstetric perspective. Noteworthy is the fact that only 44 (29.3%) respondents answered this question correctly where 75.0% of the respondents have supposedly received training and DCC was included in the curriculum. That means their knowledge of the benefits of DCC on maternal health could be lacking.

## Study’s limitations and recommendations

To the best of our knowledge, this is the first study to assess the knowledge of midwives on DCC in South Africa. The study was a small investigation into the knowledge of midwives on the benefits of DCC in a district in one of the provinces in South Africa. The results of the study can be better generalised with a larger sample size and the inclusion of more MOUs and other provinces in South Africa. This article only reports on midwives’ knowledge of the benefits of DCC. Future research should include a report on the midwives’ practices and attitudes towards DCC. The study’s sample consisted of midwives from only three MOUs in one province of South Africa. As a result, the findings may not be fully representative of midwives’ knowledge of the benefits of DCC across the country. Including a larger and more diverse sample would improve the generalisability of the results.

## Conclusion

This article gives an overview of midwives’ knowledge of the benefits of DCC. Although there are well documented advantages to the practice of DCC, the participating midwives in this research could not display their knowledge of the benefit of DCC. It is evident from the responses of this study that the midwives knew that DCC can decrease the risk of newborns staying in hospital. The midwives on the one hand had some knowledge of the increase in iron-stores and decreased respiratory distress that is associated with DCC. On the other hand, the midwives did not really associate developmental benefit of the neonate and maternal benefit with DCC. Midwives’ limited knowledge of the advantages of DCC may be a reason for the resistance to change from ICC to the recommended DCC practice.

The findings from this study could be useful in planning for the improvement of national and institutional policies and procedures on the practice of DCC with a focus on the advantages of DCC to both the mother and the newborn. The results could also be useful in the strengthening of the curriculum providing more focus on DCC during the teaching unit on the management of the stages of labour and the content of health education on the preparation of pregnant women during the antenatal care unit of the training.
